# Aldosterone levels do not predict 28-day mortality in patients treated for COVID-19 in the intensive care unit

**DOI:** 10.1038/s41598-024-58426-8

**Published:** 2024-04-03

**Authors:** Jarosław Janc, Jędrzej Jerzy Janc, Michał Suchański, Miłosz Fidut, Patrycja Leśnik

**Affiliations:** 1 Department of Anaesthesiology and Intensive Therapy, Hospital of the Ministry of the Interior and Administration, Wrocław, Poland; 2 Faculty of Medicine, Medical University of Łódź, Łódź, Poland; 3 Department of Anaesthesiology and Intensive Therapy, 4th Military Clinical Hospital, Wrocław, Poland; 4 Department of Cardiology, 4th Military Clinical Hospital, Wrocław, Poland; 5 Department of Microbiology, Wroclaw Medical University, Wrocław, Poland

**Keywords:** Biomarkers, Infectious diseases, Aldosterone, Steroid hormones

## Abstract

The immunotropic effects of aldosterone might play a role in COVID-19, as SARS-CoV-2 reportedly uses angiotensin-converting enzyme 2 receptors as an entry point into cells. Aldosterone function is closely linked to its action on mineralocorticoid receptors in kidneys; it increases the renal retention of sodium and the excretion of potassium, which increases blood pressure. Despite the large number of studies examining the effect of Ang-II and its blockers on the course of COVID-19 infection, there is still uncertainty about the role of aldosterone. The aim of the study was to assess the correlation of aldosterone, urea, creatinine, C-reactive protein (CRP), and procalcitonin (PCT) levels with 28 days of mortality in patients treated for COVID19 in an intensive care unit (ICU). This cross-selection study involved 115 adult patients who were divided into two groups: those who died within a 28-day period (n = 82) and those who survived (n = 33). The correlation of aldosterone, urea, creatinine, C-reactive protein (CRP), and procalcitonin (PCT) levels with 28 days of mortality in patients treated for COVID-19 were performed. The patients’ age, sex, scores from the APACHE II, SAPS II, and SOFA scales and comorbidities like HA, IHD and DM were also analyzed. Remarkably, the individuals who survived for 28 days were of significantly lower mean age and achieved notably lower scores on the APACHE II, SAPS II, and SOFA assessment scales. Statistically significantly higher CRP levels were observed on days 3, 5, and 7 in individuals who survived for 28 days. Creatinine levels in the same group were also statistically significantly lower on days 1, 3, and 5 than those of individuals who died within 28 days. The investigation employed both univariate and multivariate Cox proportional hazard regression models to explore factors related to mortality. In the univariate analysis, variables with a *p* value of less than 0.50 were included in the multivariate model. Age, APACHE II, SAPS II, and SOFA demonstrated significance in univariate analysis and were considered to be associated with mortality. The outcomes of the multivariate analysis indicated that age (HR = 1.03, *p* = 0.033) served as a robust predictor of mortality in the entire study population. In conclusion the plasma aldosterone level is not associated with ICU mortality in patients with COVID-19. Other factors, including the patient’s age, creatinine or CRP contribute to the severity and prognosis of the disease. This study was retrospectively registered in the Australian New Zealand Clinical Trials Registry (ANZCTR) with registration no. ACTRN12621001300864 (27/09/2021: https://www.anzctr.org.au/Trial/Registration/TrialReview.aspx?id=382563&isReview=true).

## Introduction

Aldosterone is a mineralocorticoid hormone that is produced and secreted in the zona glomerulosa of the adrenal cortex and helps regulate fluid and electrolyte balance. The stimuli needed for its biosynthesis are diverse and include the activation of the renin–angiotensin system (RAS)^1^. Aldosterone function is closely linked to its action on mineralocorticoid receptors (MR) in kidneys. It increases the renal retention of sodium and the excretion of potassium by stimulating the activity of the Na^+^–K^+^-ATPase in the basolateral membrane, which raises blood pressure^2^. Receptors for mineralocorticoid hormones can also be found on macrophages, which, when activated, might play a role in inflammation. Furthermore, MR blockade in macrophages suppresses the pro-inflammatory M1 phenotype, with beneficial effects in terms of attenuated fibrotic responses and protection from high blood pressure^3^. Studies have demonstrated that aldosterone affects the expression of inflammatory mediators as well as the final fibrosis process, given that hypertension induced by aldosterone causes cardiac inflammation with remodeling of the right and left ventricles^4–6^.

The immunotropic effects of aldosterone might play a role in coronavirus disease 2019 (COVID-19) due to the fact that severe acute respiratory syndrome coronavirus 2 (SARS-CoV-2), the pathogen responsible for COVID-19, has been demonstrated to use the angiotensin-converting enzyme 2 (ACE2) receptors as an entry point into cells^7^. ACE2 is a component of RAS and is abundantly present in lung epithelial cells. Unlike the angiotensin converting enzyme (ACE), which converts angiotensin I (Ang-I) to vasoconstrictive and pro-inflammatory angiotensin II (Ang-II), ACE2 targets Ang-II and converts it to angiotensin 1–7 (Ang 1–7), which is a vasodilator with anti-inflammatory action. As Ang-II is the major promotor of aldosterone biosynthesis, ACE II acts as an inhibitor of its production^8^. SARS-CoV-2 competes with Ang II for ACE2 in terms of internalization, which negatively influences RAS, promoting pro-inflammatory Ang-II action and might increase aldosterone production^9^. Indeed, the viral load and signs of lung injury were found to be correlated with a higher concentration of circulating Ang-II in COVID-19 patients^10^. Aldosterone controls the innate and adaptive immune response by stimulating the production of monocytes, macrophages, dentritic cells, and lymphocytes; high concentrations of aldosterone can induce potent activation of these cells and secretion of pro-inflammatory cytokines, which correlates with increased mortality and sudden death incidents in the course of Covid-19 in the geriatric patient population^11^.

There are many studies on the effect of Ang-II and its blockers on the course of COVID-19 infection; however, there is still uncertainty about the role of aldosterone^12^. Higher aldosterone levels were showed to be associated with COVID-19 severity (measured by maximal ordinal scale)^9^. While the FIDELITY Pooled Secondary Analysis showed that mineralocorticosteroid antagonist finerenon use was associated with protection against COVID-19 adverse events in patients with type 2 diabetes and chronic kidney disease^13^. Yet the data about association between aldosterone levels and COVID-19 mortality remain scarce. Given the fact that Ang-II promotes aldosterone biosynthesis, it could potentially serve as a biomarker surrogate for Ang-II to identify high-risk individuals, or it could be a new independent marker, given its pro-inflammatory and pro-fibrotic effects^11^.

**Figure 1:**
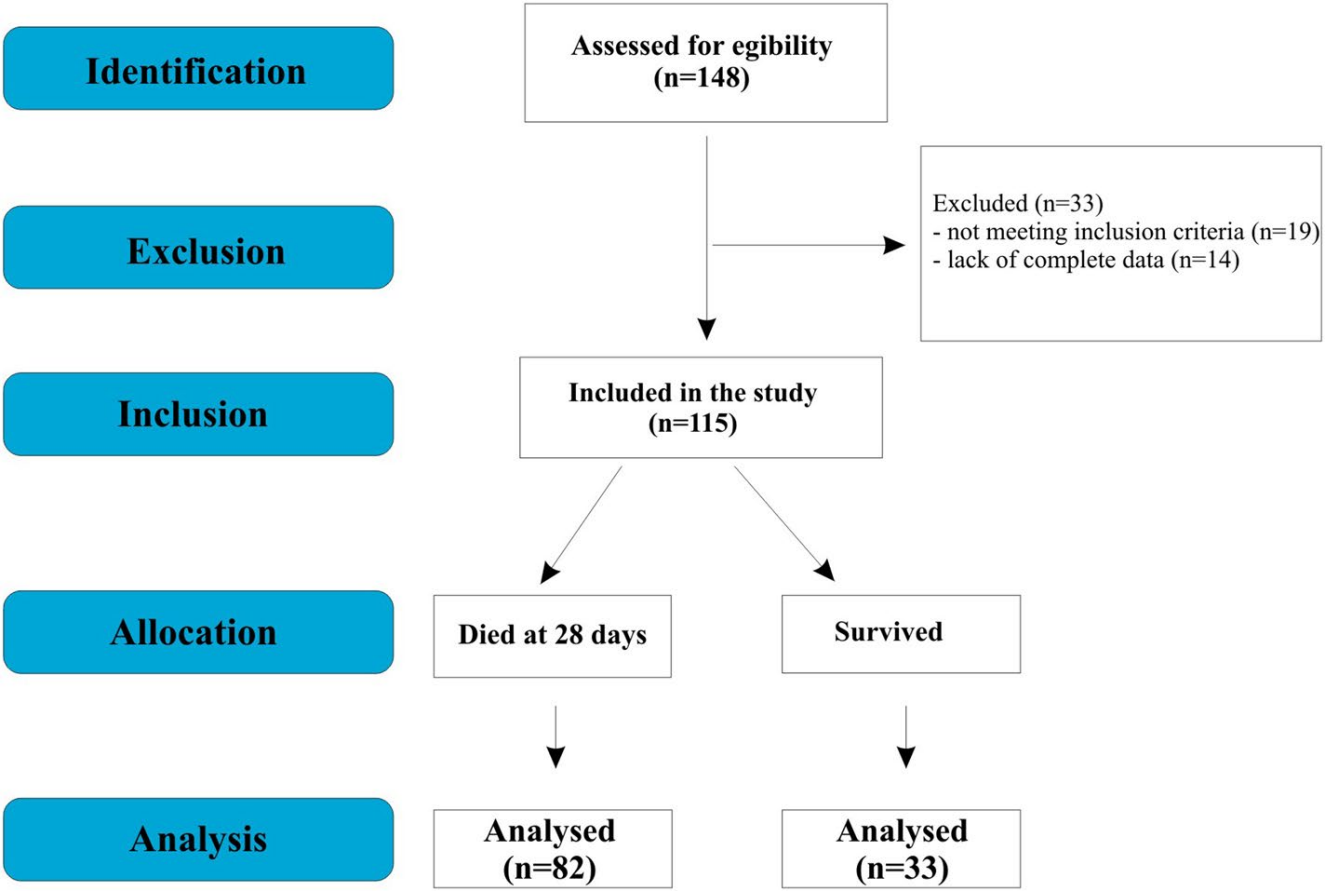
Flow-chart presenting stratification of patients participating in the study.

The aim of the study was to assess the correlation of aldosterone, urea, creatinine, C-reactive protein (CRP), and procalcitonin (PCT) levels with 28 days of mortality in patients treated for COVID-19 in the intensive care unit (Fig. 1).

## Results

### Study participants

The demographic data of the study participants are presented in Table 1. Whole group (n = 115) was divided into two groups: those who died within a 28-day period (n = 82) and those who survived for the entire duration (n = 33). The *p* value signifies the statistical significance of the disparities observed between these two groups. Remarkably, the individuals who survived for 28 days were of a significantly lower mean age (57.2 vs. 65) and achieved notably lower scores on the APACHE II (17.2 vs. 21.8), SAPS II (44.6 vs. 53.5), and SOFA (6.4 vs. 8.7) assessment scales. No statistically significant differences were found in the groups with comorbidities (AH, IHD, DM) (Table 1).

**Table 1:** Demographic data.

Variable	Dead at 28 days (n = 82)					Survived 28 days (n = 33)					*p* value
	Mean	SD	p25	Me	p75	Mean	SD	p25	Me	p75	
Age (years)	65.0	10.3	61.0	67.0	72.0	57.2	16.1	43.0	57.0	70.0	**0**.026*
BMI (kg/m^2^)	30.3	7.1	24.7	28.7	34.9	29.0	5.0	25.1	28.7	32.5	0.49*
APACHE II	21.8	9.1	16.0	20.0	25.0	17.2	8.7	11.0	16.0	21.0	**0**.012*
SAPS II	53.5	20.0	37.0	48.5	69.0	44.6	18.2	31.0	42.0	56.0	**0**.033*
SOFA	8.7	3.8	5.0	9.0	12.0	6.4	3.5	4.0	6.0	9.0	**0**.003*
Sex	Women, *n* (%) 26 (32)					Women, *n* (%)13 (40)					0.43**
	Men, *n* (%) 56 (68)					Men, *n* (%) 20 (60)					
HA	*n* (%) 49 (60)					*n* (%) 18 (55)					0.61**
	No HA, *n* (%) 33 (40)					No HA, *n* (%) 15 (45)					
IHD	*n* (%) 22 (27)					*n* (%) 12 (36)					0.31**
	No IHD, *n* (%) 60 (73)					No IHD, *n* (%) 21 (64)					
DM	*n* (%) 29 (35)					*n* (%) 11 (33)					0.84**
	No DM, *n* (%) 53 (65)					No DM, *n* (%) 22 (67)					

n, number of participants; M, mean; Me, median; p25, 25 percentile; p75, 75 percentile; p, level of statistical significance; BMI, Body Mass Index; SOFA, the Sequential Organ Failure Assessment score; APACHE II, the Acute Physiology and Chronic Health Evaluation II score; SAPS II, Simplified Acute Physiology Score II; HA, arterial hypertension; IHD, ischemic heart disease; DM, diabetes mellitus. *Mann–Whitney U test; **Chi-square test.

Significant values are in [bold].

**Figure 2:**
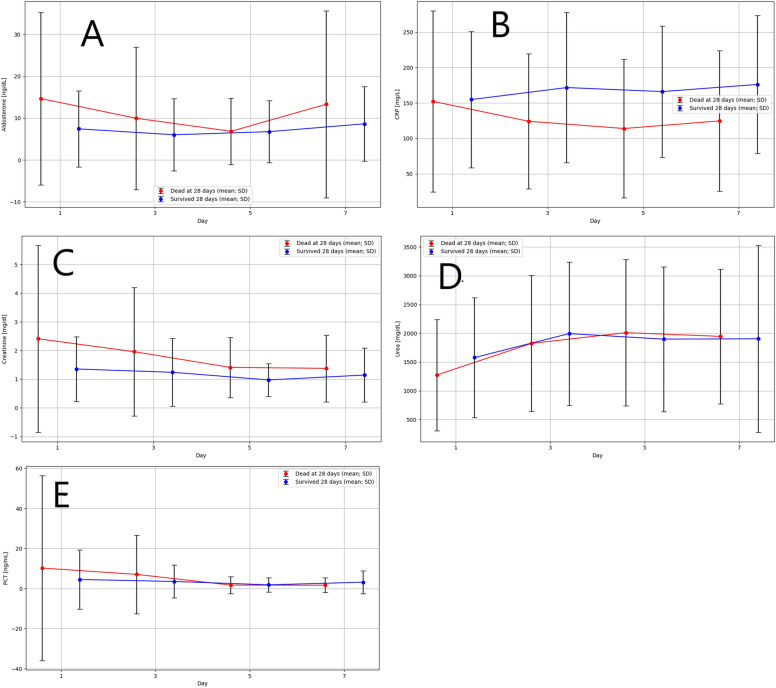
Comparison of aldosterone (**A**), CRP (**B**), creatinine (**C**), urea (**D**) and PCT (**E**) levels on day 1, day 3, day 5, and day 7 between the group of individuals who survived for 28 days and the group of individuals who died. Data presented as mean, SD.

Statistically significantly higher CRP levels were observed on days 3, 5, and 7 in individuals who survived for 28 days. Statistically significantly lower creatinine levels were observed on days 1, 3, and 5 in the survived group, compared to the group of individuals who died within 28 days (Table 2). The average results of measurements (mean and ± SD) for Aldosterone (ng/dL), CRP (mg/L), Creatinine (mg/dl), Urea (mg/dl), and PCT (ng/mL) on different days of therapy are presented in Fig. 2A–E, divided into respective groups.

**Table 2:** Comparison of selected morphological and biochemical tests between groups.

	Day	Dead at 28 days						Survived 28 days						*p* value*
		N	Mean	SD	p25	Median	p75	N	Mean	SD	p25	Median	p75	
Aldosterone (ng/dL)	1	49	14.7	20.6	3.0	5.6	19.2	24	7.4	9.1	2.6	4.0	7.1	0.15
	3	35	10.0	17.0	1.8	5.0	11.1	22	6.0	8.7	2.8	3.6	4.9	0.39
	5	30	6.9	7.9	1.9	3.5	8.7	21	6.8	7.4	2.7	4.0	6.5	0.74
	7	21	13.3	22.3	2.3	5.5	16.9	18	8.6	8.9	2.5	4.6	13.5	0.92
Urea (mg/dl)	1	80	1274.4	965.2	375.0	1310.0	1995.0	33	1578.1	1039.3	700.0	1635.0	2100.0	0.15
	3	68	1823.4	1179.3	1100.0	1930.0	2600.0	33	1993.9	1244.6	1330.0	2150.0	2580.0	0.62
	5	57	2010.1	1272.0	1200.0	1950.0	2700.0	32	1898.1	1256.9	820.0	2085.0	2700.0	0.79
	7	47	1946.0	1168.6	1080.0	1900.0	2600.0	31	1904.2	1623.8	10.0	1850.0	3230.0	0.84
CRP (mg/L)	1	82	152.2	127.9	50.0	129.0	195.0	33	154.8	95.9	77.1	157.0	215.0	0.48
	3	66	124.2	95.4	55.0	99.0	176.0	33	171.7	105.9	99.4	140.0	212.0	**0**.**013**
	5	55	114.0	97.6	38.0	77.0	169.0	32	166.1	92.4	88.5	164.5	228.5	**0**.**008**
	7	46	124.7	99.1	55.4	100.5	197.0	31	176.2	97.2	108.0	170.0	231.3	**0**.**015**
Creatinine (mg/dl)	1	82	2.4	3.3	0.8	1.1	2.7	33	1.4	1.1	0.7	0.8	1.5	**0**.**049**
	3	68	2.0	2.2	0.7	1.0	2.1	33	1.2	1.2	0.6	0.8	1.2	**0**.**041**
	5	57	1.4	1.0	0.7	0.9	1.8	32	1.0	0.6	0.6	0.7	1.2	**0**.**021**
	7	48	1.4	1.2	0.7	0.8	1.6	30	1.1	0.9	0.6	0.8	1.6	0.30
PCT (ng/mL)	1	80	10.2	46.1	0.2	0.6	1.6	32	4.5	14.8	0.1	0.6	1.9	0.81
	3	65	7.0	19.6	0.1	0.5	1.6	30	3.5	8.2	0.2	0.5	2.5	0.47
	5	55	1.7	4.3	0.1	0.4	0.9	30	1.8	3.6	0.1	0.5	1.3	0.40
	7	44	1.6	3.7	0.1	0.4	1.3	30	3.1	5.7	0.1	0.8	2.2	0.20

n, number of participants; M, mean; Me, median; p25, 25 percentiles; p75, 75 percentiles; p, level of statistical significance; CRP, C-reactive protein; PCT, procalcitonin; *Mann–Whitney U test.

Significant values are in [bold].

### Mortality

**Figure 3:**
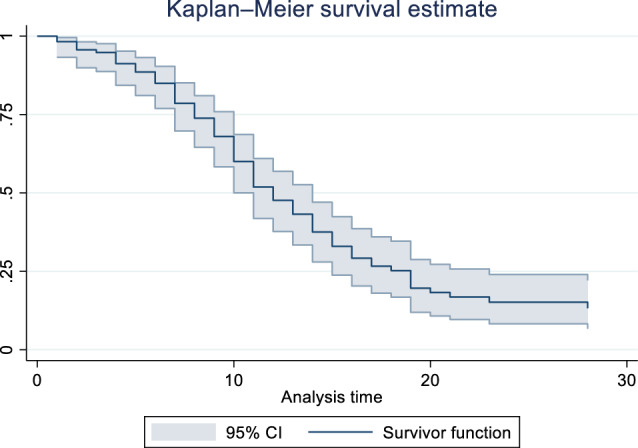
Kaplan–Meier survival curves.

Kaplan–Meier survival estimate is presented in Fig. 3. Of the total number of valid observations (115 patients), 82 (71.30%) died, and 33 (28.70%) patients survived for 28 days. The median survival was 12 days (the 25th percentile was 8 days, and the 75th percentile was 18 days).

### Independent predictors of outcomes

The investigation employed both univariate and multivariate Cox proportional hazard regression models to explore factors related to mortality. In the univariate analysis, variables with a *p* value of less than 0.50 were included in the multivariate model. Factors that demonstrated significance in both univariate and multivariate analyses were considered to be associated with mortality, as depicted in Table 3.

**Table 3:** Univariate and multivariate Cox regression analysis of risk factors influence on mortality.

Variables	HR	95% CI		*p* value
	Univariate model			
Age	**1**.**03**	**1**.**01**	**1**.**04**	**0**.**004**
BMI	1.02	0.99	1.06	0.214
APACHE II	**1**.**03**	**1**.**01**	**1**.**05**	**0**.**015**
SAPS II	**1**.**02**	**1**.**01**	**1**.**03**	**0**.**003**
SOFA	**1**.**10**	**1**.**04**	**1**.**16**	**0**.**001**
ALDOSTERONE (ng/dL)	1.01	1.00	1.03	0.097
Urea (mg/dl)	1.00	1.00	1.00	0.320
CRP (mg/L)	1.00	1.00	1.00	0.903
Creatinine (mg/dl)	**1**.**11**	**1**.**03**	**1**.**19**	**0**.**005**
PCT (ng/mL)	1.00	1.00	1.01	0.132
SEX (ref. M)	0.81	0.51	1.29	0.377
HA (ref. No)	1.02	0.66	1.59	0.919
IHD (ref. No)	0.83	0.51	1.35	0.454
DM (ref. No)	1.28	0.81	2.02	0.290
	Multivariate model			
Age	**1**.**03**	**1**.**01**	**1**.**03**	**0**.**033**

HR, hazard ratio; CI, confidence interval; BMI, Body Mass Index; SOFA, the Sequential Organ Failure Assessment score; APACHE II, the Acute Physiology and Chronic Health Evaluation II score; SAPS II, Simplified Acute Physiology Score II; CRP, C-reactive protein; PCT, procalcitonin; HA, arterial hypertension; IHD, ischemic heart disease; DM, diabetes mellitus.

Significant values are in [bold].

The outcomes of the multivariate analysis indicated that only AGE (HR = 1.03, *p* = 0.033) served as a robust predictor of mortality in the entire study population. Multivariate analysis of the remaining parameters did not show statistical significance.

## Discussion

In this study, we attempted to find an association between the serum aldosterone levels measured on days 1, 3, 5, and 7 and the overall survival in the intensive care unit (ICU) for a duration of 28 days. Even though the mean plasma aldosterone levels were higher in the patients who did not survive for 28 days, the results were not statistically significant, indicating that aldosterone is not associated with ICU mortality. A previous study by Villard et al.^14^ reported a worse disease course in patients with higher aldosterone levels at admission; specifically, those with higher plasma aldosterone levels were more likely to be admitted to the ICU. This might indicate that plasma aldosterone levels might be associated with the rate of admissions to ICUs but do not correlate with overall days survived in the ICU. Studies suggest that SARS-CoV-2, by competing with angiotensin II for the ACE2 receptor, indirectly increases Ang-II levels, which should increase aldosterone biosynthesis. It is possible that aldosterone production in COVID-19 patients increases with disease severity and reaches a peak when the state of the patient calls for an ICU admission. More research is needed to reach a definite conclusion.

Systemic inflammation evoked by SARS-CoV-2 is a hallmark of COVID-19, and one of the most widely used biomarkers for inflammation is an acute-phase protein CRP, which is biosynthesized in the liver in response to elevated interleukin-6 levels^14,15^. Interestingly, our results showed statistically significantly lower levels of serum CRP in patients who died during their 28-day stay in the ICU, yet it was not associated with higher mortality. This outcome contradicts previous findings indicating a relationship between CRP serum concentration and severity of the disease^16–20^. This discrepancy could be a result of the small size of the group that survived beyond 28 days.

Higher serum creatinine levels have been shown to be another strong predictor of ICU admission and mortality^21^. Our study provides additional evidence that high serum creatinine concentration is a predictor of COVID-related ICU mortality; higher creatinine levels were observed on days 1, 3, and 5 in patients who died within 28 days than in the group of individuals who survived. Many large studies, including meta-analyses, have confirmed the association of high creatinine levels with increased mortality in severely ill patients^21–23^.

In our study, univariate analysis showed increased mortality in the group of patients with higher APACHE II, SAPS II, and SOFA scores. Multivariate analysis showed that age (HR = 1.03, *p* = 0.033) was a robust predictor of mortality in the entire study population, similar to the finding of the meta-analysis by Kowsar et al.^24^. COVID-19 is a disease with a difficult-to-predict course. In intensive care units, numerous scales are routinely used to assess the risk of death and estimate the severity of the condition and organ function upon admission. Our study selected the most frequently used and valuable ICU scales. In a study by Monk et al., no advantage of any mortality scoring systems applied to COVID-19 was demonstrated. The study showed that SOFA, SAPS II, APACHE II, and ISARIC 4-C scores accurately predicted mortality in critically ill patients with COVID-19. The SOFA score executed the best. The study conducted by our team showed correlations of the values obtained in the SOFA, APACHE II, and SAPS II scales with mortality^12^.

Comorbidities associated with higher mortality following SARS-CoV-2 infection are hypertension and diabetes. There is a strong association between in-hospital mortality due to COVID-19 and hypertension, coronary heart disease, and diabetes^25^, although our study did not show a statistically significant effect of these factors on mortality. Researchers have postulated that the use of RAS blockers, such as ACE inhibitors or angiotensin receptor blockers (ARB), which are frequently administered in these conditions, might contribute to upregulation of ACE2, which could potentially promote cell entry of SARS-CoV-2, causing worse outcomes. The BRACE CORONA clinical trial later proved that neither continuation nor discontinuation of these drugs had a significant impact on mortality or COVID-19 progression^26^. Mineralocorticoid antagonists have also failed to show any effect on mortality in a recent meta-analysis^27^. The activation of RAAS by SARS-CoV-2 may lead to a direct increase in aldosterone production; one of the production sites may be the endothelial cells of the pulmonary vessels^29^. Higher levels of aldosterone may induce severe forms of COVID-19, especially in older patients, by promoting the inflammatory response and inducing electrolyte disorders such as hypokalemia.

One of the treatment options for COVID-19, in the case of high aldosterone concentration correlation with mortality, could be the use of mineralocorticosteroid receptor antagonists (MRAs). Reports on the effect of MRAs on COVID-19 are ambiguous^22,28,29^. MRAs, ACE inhibitors (ACE-I), and angiotensin receptor blockers (ARBs) were analyzed for their effects on COVID-19. Many patients discontinued RAASi treatment during the first phase of the COVID-19 pandemic due to the potential for these drugs to increase ACE 2 expression and levels. Numerous studies have shown that RAASi use is not linked to the risk of COVID-19^28–30^. Compared to ACE-I and ARBs, using MRAs in COVID-19 may provide some benefits. Additionally, MRAs, by stimulating ADAM metalloproteinase domain 17 protein, increases circulating ACE 2, which might bind SARS-CoV-2 as a competitive interceptor^29^. MRAs can suppress the expression of type II transmembrane serine protease TMPRSS2, which increases viral uptake of SARS-CoV-2 in target cells by promoting membrane fusion of Spike glycoprotein through a proteolytic cleavage between the S1 and S2 subunits and, also, by cleaving ACE 2 that, in turn, activates cathepsin L-dependent pathway^31^.

Inhibiting these pathways induced by MRAs could suppress or reduce viral entry in human cells. It might benefit COVID-19 infection and acute respiratory distress syndrome^32,33^. The reason why aldosterone levels were measured in our ward was to investigate the correlation of its level with mortality, which could potentially result in appropriate treatment. The result of our study did not justify the rationale for use of MRAs in the treatment of Covid-19 patients.

This study has certain limitations that need to be considered when interpreting the findings. First, this is a single-center study with a small simple size. Second, it is a retrospective, cross-selection study; thus, further prospective studies should verify the findings. Further, the chemiluminescent immunoassay method may not reveal aldosterone serum levels. Wiegand et al.^34^ demonstrated that “aldosterone cannot be accurately estimated in serum from patients with SARS-CoV-2 infection using direct competitive immunoassay. When measured using gold-standard LCMSMS, serum aldosterone is found to be remarkably low in most patients with COVID-19”. However, the chemiluminescent immunoassay method used for aldosterone determination in our study is a well-validated measurement method that is routinely used for aldosterone level determinations in laboratory practice. The method used in the study was compared with a manual radioimmunoassay (RIA), in accordance with the guidelines of CLSI EP9 (Clinical & Laboratory Standards Institute—Measurement Procedure Comparison and Bias Estimation). The correlation coefficients were 0.98 (for serum) and 0.90 (for urine). In addition, this parameter is subject to monthly international control (RIQAS external quality control system run by Randox).

## Conclusion

The plasma aldosterone level is not associated with ICU mortality in patients with COVID-19. Other factors, including the patient’s age, creatinine and CRP contribute to the severity and prognosis of the disease.

## Methods

### Study participants

The stratification of patients participating in the study is shown in the Flow-chart (Fig. 1). The single-center cross-selection study was conducted from September 2020 to September 2022 at the Intensive Care Unit 4 Military Hospital of Wroclaw, Poland. A group of 115 adult patients (irrespective of gender) who were treated in the intensive care unit (ICU) of the 4th Military Clinical Hospital in Wroclaw during the period from September 2020 to September 2022 were enrolled in the present study. All the patients were treated for SARS-CoV-2 infection. Patients under 18 years of age and with negative SARS-CoV-2 test were excluded from the study.

### Outcomes

The study aimed to assess the correlation of aldosterone, urea, creatinine, CRP, and PCT levels with 28 days of mortality in patients treated for COVID-19 in the intensive care unit. The secondary objective was to assess whether aldosterone levels could serve as a marker of the severity of SARS-CoV-2 infection in patients treated in the ICU.

### Measures

Demographic data (age, sex, and BMI) were included in the study data. Aldosterone, PCT, and CRP were determined in the blood samples collected via venous cannula upon hospital admission and on the 1st, 3rd, 5th, and 7th days of hospital stay. Further, the Acute Physiology and Chronic Health Evaluation (APACHE II) and Simplified Acute Physiology Score (SAPS II) scales were used upon admission, and the Sequential Organ Failure Assessment (SOFA) score was assessed each day. The impact of comorbidities such as hypertension (AH), ischemic heart disease (IHD) and diabetes (DM) was taken into account in the analysis.

### Ethics

The study protocol was approved by the Bioethics Committee at the Military Medical Chamber in Warsaw, Poland (approval no.: KB–3/21, approval date: 21.05.2021). The study was carried out in accordance with the guidelines of the Declaration of Helsinki and Good Clinical Practice. Informed and written consent was obtained from all patients. The study was registered in the Australian New Zealand Clinical Trials Registry (ANZCTR) with registration no. ACTRN12621001300864. The standards for Strengthening the Reporting of Observational Studies in Epidemiology (STROBE) were followed.

### Simple size

The sample size analysis was conducted based on one of the primary objectives, in which it was assumed that a higher level of aldosterone would be observed in the group of individuals who had died. The minimum sample size required to detect this difference, assuming alpha = 5%, power = 80%, and confidence level (CI) = 95%, was 99 patients in total. Additionally, a data completeness risk of 10% was taken into account. The final sample size was 115 participants. The sample size analysis was performed using G * Power software.

### Statistical analysis

The calculations were conducted using STATA v. 17 software (StataCorp, 2021; Stata Statistical Software: Release 17; College Station, TX: StataCorp LLC, USA). The categorical variables were presented as frequencies (n) and percentages (%). A chi-squared test was applied to assess the association between categorical variables. The quantitative variables were presented in tables as mean, standard deviation (SD), median (Me) values, and quartiles (p25, p75). The significance of the mean differences between groups was determined using the Mann–Whitney U test. Overall survival (OS) was analyzed using the Kaplan–Meier method. Multivariate analysis was performed using the Cox proportional hazard regression model. A *p* value below 0.05 was considered statistically significant.

## Data Availability

The datasets generated during and/or analyzed during the current study are available from the corresponding author on reasonable request.

## References

[1] Boulkroun S, Fernandes-Rosa FL, Zennaro M-C (2020). Old and new genes in primary aldosteronism. Best Pract. Res. Clin. Endocrinol. Metab..

[2] Palmer BF (2015). Regulation of potassium homeostasis. Clin. J. Am. Soc. Nephrol..

[3] Marzolla V (2014). Mineralocorticoid receptor in adipocytes and macrophages: A promising target to fight metabolic syndrome. Steroids.

[4] Ferreira NS, Tostes RC, Paradis P, Schiffrin EL (2021). Aldosterone, inflammation, immune system, and hypertension. Am. J. Hypertens..

[5] Weber KT (2001). Aldosterone in congestive heart failure. N. Engl. J. Med..

[6] Weber KT, Brilla CG (1991). Pathological hypertrophy and cardiac interstitium. Fibrosis and renin-angiotensin-aldosterone system. Circulation.

[7] Li W (2003). Angiotensin-converting enzyme 2 is a functional receptor for the SARS coronavirus. Nature.

[8] Bourgonje AR (2020). Angiotensin-converting enzyme 2 (ACE2), SARS-CoV-2 and the pathophysiology of coronavirus disease 2019 (COVID-19). J. Pathol..

[9] Beyerstedt S, Casaro EB, Rangel ÉB (2021). COVID-19: Angiotensin-converting enzyme 2 (ACE2) expression and tissue susceptibility to SARS-CoV-2 infection. Eur. J. Clin. Microbiol. Infect. Dis..

[10] Shukla AK, Banerjee M (2021). Angiotensin-converting-enzyme 2 and renin-angiotensin system inhibitors in COVID-19: An update. High Blood Press Cardiovasc. Prev..

[11] Campana P (2022). The elderly at risk: Aldosterone as modulator of the immune response to SARS-CoV-2 infection. Geroscience.

[12] Monk M (2023). A comparison of ICU mortality scoring systems applied to COVID-19. Cureus.

[13] Pitt B (2022). Association of finerenone use with reduction in treatment-emergent pneumonia and COVID-19 adverse events among patients with type 2 diabetes and chronic kidney disease: A FIDELITY pooled secondary analysis. JAMA Netw. Open.

[14] Villard O (2020). The plasmatic aldosterone and C-reactive protein levels, and the severity of Covid-19: The Dyhor-19 study. J. Clin. Med..

[15] Petrilli CM (2020). Factors associated with hospital admission and critical illness among 5279 people with coronavirus disease 2019 in New York City: Prospective cohort study. BMJ.

[16] Morley JJ, Kushner I (1982). Serum C-reactive protein levels in disease. Ann. N. Y. Acad. Sci..

[17] Zhang J, Yu M, Tong S, Liu L-Y, Tang L-V (2020). Predictive factors for disease progression in hospitalized patients with coronavirus disease 2019 in Wuhan, China. J. Clin. Virol..

[18] Cariou B (2020). Phenotypic characteristics and prognosis of inpatients with COVID-19 and diabetes: The CORONADO study. Diabetologia.

[19] Zhang X (2020). Viral and host factors related to the clinical outcome of COVID-19. Nature.

[20] Ali N (2020). Elevated level of C-reactive protein may be an early marker to predict risk for severity of COVID-19. J. Med. Virol..

[21] Smilowitz NR (2021). C-reactive protein and clinical outcomes in patients with COVID-19. Eur. Heart J..

[22] Komaru Y, Doi K (2021). Does a slight change in serum creatinine matter in coronavirus disease 2019 (COVID-19) patients?. Kidney Res. Clin. Pract..

[23] Al-Aly Z, Balasubramanian S, McDonald JR, Scherrer JF, O’Hare AM (2012). Greater variability in kidney function is associated with an increased risk of death. Kidney Int..

[24] Kowsar R (2023). Risk of mortality in COVID-19 patients: A meta- and network analysis. Sci. Rep..

[25] Tsampasian V, Corballis N, Vassiliou VS (2022). Renin-angiotensin-aldosterone inhibitors and COVID-19 infection. Curr. Hypertens. Rep..

[26] Krieger EM (2018). Spironolactone versus clonidine as a fourth-drug therapy for resistant hypertension: The ReHOT randomized study (Resistant Hypertension Optimal Treatment). Hypertension.

[27] Kim J, Miyazaki K, Shah P, Kozai L, Kewcharoen J (2022). Association between mineralocorticoid receptor antagonist and mortality in SARS-CoV-2 patients: A systematic review and meta-analysis. Healthcare.

[28] Vaduganathan M (2020). Renin-angiotensin-aldosterone system inhibitors in patients with Covid-19. N. Engl. J. Med..

[29] Savarese G, Benson L, Sundström J, Lund LH (2021). Association between renin-angiotensin-aldosterone system inhibitor use and COVID-19 hospitalization and death: A 1.4 million patient nationwide registry analysis. Eur. J. Heart Fail..

[30] Mancia G, Rea F, Ludergnani M, Apolone G, Corrao G (2020). Renin-angiotensin-aldosterone system blockers and the risk of Covid-19. N. Engl. J. Med..

[31] Zipeto D, da Fonseca Palmeira J, Argañaraz GA, Argañaraz ER (2020). ACE2/ADAM17/TMPRSS2 interplay may be the main risk factor for COVID-19. Front. Immunol..

[32] Campana P, Flocco V, Aruta F, Cacciatore F, Abete P (2021). Can aldosterone increase interleukin-6 levels in Covid-19 pneumonia?. J. Med. Virol..

[33] Vardhana SA, Wolchok JD (2020). The many faces of the anti-COVID immune response. J. Exp. Med..

[34] Wiegand M (2022). Unquantifiably low aldosterone concentrations are prevalent in hospitalised COVID-19 patients but may not be revealed by chemiluminescent immunoassay. Endocr. Connect..

